# Robotic Capsule Endoscopy: Simultaneous Gastric and Enteric Evaluation in Real-World Practice

**DOI:** 10.3390/diagnostics16020334

**Published:** 2026-01-20

**Authors:** Hélder Cardoso, Miguel Mascarenhas, Joana Mota, Miguel Martins, Maria João Almeida, Joana Frias, Catarina Cardoso Araújo, Francisco Mendes, Margarida Marques, Patrícia Andrade, Guilherme Macedo

**Affiliations:** 1 Department of Gastroenterology, São João University Hospital, 4200-319 Porto, Portugal; miguelmascarenhassaraiva@gmail.com (M.M.); guilhermemacedo59@gmail.com (G.M.); 2 WGO Porto Gastroenterology and Hepatology Training Center, 4200-319 Porto, Portugal; 3 Faculty of Medicine, University of Porto, 4200-319 Porto, Portugal

**Keywords:** capsule endoscopy, gastroscopy, robotics, magnetic fields, small intestine, Crohn disease, angiodysplasia, polyps

## Abstract

**Background/Objectives**: Robotic capsule endoscopy (RCE) is an emerging technology that combines magnetically controlled gastric navigation with conventional capsule enteroscopy (CE), enabling a minimally invasive, comprehensive evaluation of the upper- and mid-gastrointestinal tract. This study aimed to characterize the real-world implementation and diagnostic performance of RCE in a European tertiary referral center. **Methods**: A retrospective, single-center analysis was conducted on adult patients (≥18 years) who underwent RCE (Omom RC) between June 2023 and July 2025. Eligible patients had a clinical indication for small bowel CE and a concurrent requirement for diagnostic gastroscopy or reassessment of known gastric lesions. The RCE protocol comprised an initial robotic-guided gastric examination followed by passive transit through the small bowel. **Results**: A total of 85 patients were included (52% female), with a median age of 49 years (IQR 40–64). The most common indications were suspected or established inflammatory bowel disease (57%) and iron deficiency anemia (31%). Gastric preparation was rated at least fair in 98% of cases, with good preparation in 38%. Median gastric transit time was 74 min (IQR 35–106). Relevant gastric findings were identified in 39 cases (46%), namely polyps (18%) and angiectasias (8%, including one with active bleeding), in addition to signs of chronic gastritis. Thirteen patients underwent subsequent endoscopy, resulting in seven therapeutic procedures. Small bowel findings were present in 60 patients (71%), including P3 (active bleeding) in 3% and P2 lesions (angiectasias, ulcers, tumors, varices) in 39%. One moderate adverse event occurred: small bowel capsule retention in a patient with multifocal neuroendocrine tumor and ileostomy, requiring endoscopic intervention. **Conclusions**: Robotic capsule endoscopy is a feasible tool for dual-region gastrointestinal evaluation. It enables high-quality gastric visualization, facilitates early detection of clinically actionable lesions, and maintains the diagnostic yield expected from standard small bowel CE. These findings support the integration of RCE into diagnostic pathways for patients requiring simultaneous gastric and small bowel assessment.

## 1. Introduction

### 1.1. Capsule Endoscopy Evolution

The development of capsule endoscopy (CE), at the beginning of this century, revolutionized small bowel endoscopy surpassing previous constraints and enabling a minimally invasive, mucosa-level, thorough visualization [1].

Its following dissemination in clinical practice led to a widening of its clinical scope beyond recurrent gastrointestinal bleeding, dramatically transforming the diagnosis and management of digestive tract disorders. Conventional CE became the gold standard for evaluating obscure gastrointestinal bleeding, small bowel Crohn’s disease, small bowel tumors, and iron deficiency anemia [2,3,4].

Consecutive technological improvements have been implemented, especially related to image resolution, camera viewing angle, and frame rate. A major advancement was achieved with the introduction of a double-headed camera for colon capsule endoscopy (CCE). Designed for the detection of colon pathology, it also allowed for enhanced visualization of the upper gastrointestinal mucosa [5,6].

However, CE has limitations in the gastric evaluation due to its passive movement, lack of insufflation, and erratic gastric transit, often leading to poor mucosal visualization.

### 1.2. Limitations of Conventional Capsule Endoscopy

A comprehensive assessment of the gastric mucosa has long been a challenge for CE. The challenging anatomy, which is quite different from that of the small bowel, and the lack of controlled movement impair targeted visualization of gastric landmarks (cardia, body, fundus, incisura, and antrum). As such, the diagnostic yield of conventional CE in the stomach is inevitably lower compared to the current gold standard, esophagogastroduodenoscopy (EGD) [6,7].

For multiple clinical conditions, a sequential approach of endoscopic procedures (EGD followed by CE) is required; even so, EGD is most often diagnostic, without therapeutic intervention. Necessarily, this implies increasing cost, burden, and time [2,8].

### 1.3. Robotic Capsule Endoscopy Overview

To overcome this shortcoming of CE, current research mainly focuses on external magnetic field maneuvering, namely robotic capsule endoscopy (RCE).

A robotic arm with a permanent magnet enables control of the movement of the magnet-incorporated, single-camera RCE in real time and is employed upon reaching the stomach. This system allows for active, operator-guided navigation through the stomach before passive transit into the small bowel [7,9].

Successive advancements enabled the deployment of a self-operating RCE system with automated gastric guidance without manual control. This technology combines the navigational advantages of EGD with the minimally invasive nature of CE, optimizing both gastric and enteric diagnostics, as well as patient comfort [9,10].

### 1.4. Rationale and Objectives of the Study

Despite growing interest in RCE, real-world data remains limited, particularly in Western populations [7,11].

This study aims to describe the clinical performance and safety of RCE in the dual diagnostic context of gastroscopy and enteroscopy.

The primary objective is to evaluate the feasibility and lesion detection of RCE for combined assessment of the gastric and small bowel mucosa.

The secondary objectives are to assess the quality of stomach mucosa visualization, prevalence of actionable findings, follow-up interventions, and adverse events.

## 2. Materials and Methods

### 2.1. Study Design and Population

A retrospective, single-center, observational cohort study was conducted in a Portuguese tertiary academic center. The study included adult patients (≥18 years) that performed RCE between June 2023 and July 2025.

The inclusion criteria comprised patients with a conventional indication for small bowel CE and a concurrent requirement for gastric assessment. The indication for gastric assessment involved pathologies such as chronic gastritis, Crohn’s disease, cancer screening, and surveillance of angiectasias or polyps.

The exclusion criteria consisted of pregnancy, suspected digestive tract obstruction with negative patency test, large hiatal hernia, history of gastric surgery, and pacemakers or metallic implants contraindicating magnetic use.

Small bowel findings were labeled according to the Saurin classification of bleeding potential of lesions [12].

### 2.2. Description of RCE System and Protocol

The RCE equipment includes the Robotic Movement Unit, the Control Console, and the Robotic Capsule with Recording Unit (Omom, Jinshan, Chongqing, China). The RCE movement system involves upper and lower magnetic fields located in the robotic arm and examination bed that interact to control the capsule motion. The Control Console has the software for real-time visualization and control of the gastric examination, either in automatic or manual mode. The Robotic Capsule (Omom RC, Jinshan, Chongqing, China) delivers image acquisition with a high-definition camera, a wide viewing angle of 172°, and up to 10 frames per second [10,13].

The patient workflow, according to the manufacturer instructions, starts with overnight fasting and initial gastric preparation with water loading (1–1.5 L with simethicone). After RCE activation and swallowing, the gastric phase is enabled in automatic mode or manual mode using a joystick-controlled robotic arm. In this study, the automatic mode was employed in all patients. During the gastric examination, the patient remains in the supine position. Afterwards, the capsule naturally advances through the pylorus into the duodenum. If gastric transit time exceeds one hour, a prokinetic drug is used (domperidone 10 mg tablet). Finally, the enteric phase is similar to conventional CE, with standard passive small bowel transit and imaging [10,13,14].

Concerning tolerance, all procedures are performed without sedation. During the gastric examination, there is no need for the patient to change position. Patient comfort and adverse events are recorded.

### 2.3. Data Collection Parameters

Relevant clinical parameters collected include demographics related to age, sex, and comorbidities; clinical indication for CE and gastric assessment; gastric and enteric lesion types and locations; interventions triggered by the RCE findings; and adverse events. Adverse events were graded with the ASGE lexicon and the AGREE classification [15].

Procedure data recorded include the RCE date, quality of gastric preparation (graded as poor/fair/good), enteric preparation (Brotz score) [16], gastric transit time, small bowel transit time, and technical failure.

### 2.4. Statistical Analysis

The primary endpoints evaluated were lesion detection in the stomach and the small bowel detection rate. Subgroup analysis was performed according to demographics and clinical indication.

Regarding descriptive statistics, continuous variables are expressed as the median with interquartile range (IQR, P25–P75), and categorical variables are presented as counts and percentages. Comparisons between variables are performed using Fisher’s exact test, chi-square test, Mann–Whitney test, or Kruskal–Wallis test, as appropriate, with significance defined as *p* < 0.05. SPSS version 28.0 was used to perform the statistical analysis.

## 3. Results

### 3.1. Baseline Characteristics

A total of 85 patients who underwent RCE were included, with a median age of 49 years (IQR 40–64) and an equivalent sex distribution (Table 1). Some patients had a history of digestive tract surgery, namely segmental enterectomy (7%) and colectomy (5%).

The main clinical indications for small bowel CE were suspected Crohn’s disease (35%), iron deficiency anemia (31%), and Crohn’s disease surveillance (22%). Other indications included surveillance of small bowel tumors (5%—neuroendocrine, lymphoma, and gastrointestinal stromal tumor).

Patients with Crohn’s disease were younger than those referred due to iron deficiency anemia (median 45 vs. 65 years; *p* < 0.001).

The main purpose for combined CE gastroscopy comprised Crohn’s disease, either clinically suspected (25%) or under assessment (20%), followed by investigation of dyspepsia (9%), surveillance of gastric polyps (9%), chronic gastritis (8%), angiectasias (7%), investigation of iron deficiency anemia (8%), cancer screening (8%), and polyposis syndromes (5%).

### 3.2. Gastric Assessment

The median gastric transit time was 74 min and over 10 min in most patients (86%), comparable to conventional CE procedure.

The overall gastric mucosa visualization was labeled as fair or better in nearly all exams (98%) and good in a major portion (38%).

**Figure 1:**
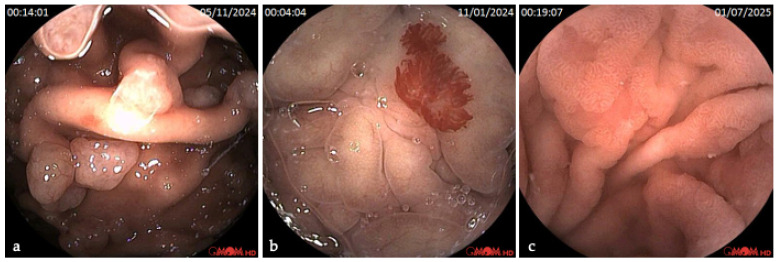
Images of gastric mucosa lesions in RCE. (**a**) Gastric polyps; (**b**) gastric angiectasia; (**c**) gastritis with intestinal metaplasia.

There were gastric findings detected in 46% of the RCE exams (Figure 1). The most common were gastritis (19%), polyps (18%), and angiectasias (8%), including gastric vascular ectasia (1%) and bleeding (1%). Structural anomalies were seldom detected, such as diverticulum (1%) and subepithelial lesion (1%). The distribution by anatomical regions is presented in Table 2.

Patients with angiectasia detection were older than those without lesions (median 71 vs. 45 years; *p* < 0.001). Concerning the main clinical indications for CE, patients referred with anemia presented a higher gastric lesion detection rate (84% vs. 30%; *p* < 0.001).

### 3.3. Small Bowel Findings

The median enteric transit time was 5 h and 7 min, similar to conventional CE.

Overall small bowel preparation was labeled as fair or better in most exams (95%), and good in a lesser portion (11%).

**Figure 2:**
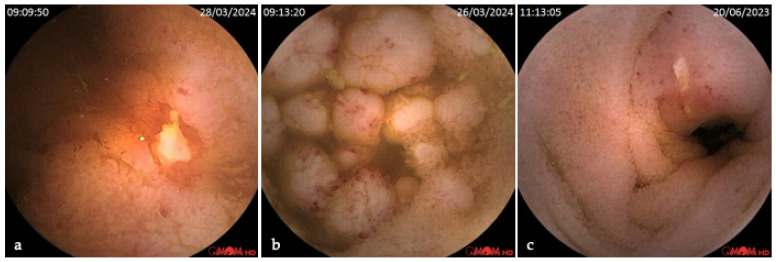
Images of small bowel mucosa lesions in RCE. (**a**) Crohn’s disease ulcer; (**b**) follicular lymphoma; (**c**) neuroendocrine tumor.

Regarding enteroscopy, relevant findings were present in most (71%) patients (Figure 2). These included P3 (active bleeding, 4%), P2 ulcers (18%), P2 angiectasias (16%), P2 tumors (4%), P2 varices (2%), P1 red spots (25%), P1 erosions (13%), and P1 polyps (5%).

### 3.4. Follow-Up and Interventions

These RCE gastric findings triggered further procedures in several situations, particularly endoscopy therapy, and triggered changes in patient management.

Thirteen patients underwent follow-up EGD, with argon plasma ablation therapy in four cases and polypectomy in three cases.

Several findings led to adjustments in medical therapy related to Crohn’s disease, chronic gastritis, and anemia, as well as the surveillance of identified gastric lesions.

### 3.5. Safety Outcomes

During this study, no magnetic field-related complications were detected.

There was one moderate adverse event (Grade IIIa): small bowel capsule retention occurred in a patient with multiple enteric neuroendocrine tumors and a terminal ileostomy, requiring endoscopic intervention. No other significant discomfort or intolerance related to RCE was reported.

## 4. Discussion

To the best of our knowledge, this is the largest study reporting combined gastrointestinal assessment with RCE in a Western country.

There was a high visualization adequacy of the gastric mucosa, rated as at least fair in almost all cases, which was associated with considerable diagnostic performance. RCE enabled simultaneous thorough assessment of the gastric and small bowel mucosa in a single session. This allowed enhanced diagnostic efficiency, especially in patients with overlapping upper- and mid-gastrointestinal symptoms.

Being minimally invasive, RCE does not permit tissue sampling and is contingent upon adequate gastric preparation quality and minimal gastric motility, albeit the external magnetic field maneuvering. Indeed, correct execution of operator-guided navigation requires trained personnel for robotic control and corresponding interpretation.

This innovative technique was demonstrated to be beneficial in different clinical settings, especially in the evaluation of standard indications for CE, such as inflammatory bowel disease, iron deficiency anemia, and hereditary polyposis syndromes. Likewise, it was useful for surveillance of known gastric lesions, including assessment of treatment response after polypectomy or vascular lesion ablation. Concerning the detection of gastric lesions, RCE helped identify which patients require follow-up EGD for targeted biopsies or therapeutic procedures.

From this standpoint, RCE has the potential to reduce the requirement for multiple invasive procedures. Nonetheless, due to the retrospective design of the study, it was not possible to assess other performance parameters, namely sensitivity, specificity, negative predictive value, or non-inferiority margins, as most patients did not perform EGD in a close time frame. To accurately assess the diagnostic yield of RCE in the gastric mucosa, subsequent prospective studies are required, preferably with same-day EGD in a blinded design.

When compared with the gold standard EGD, RCE has the advantage of being minimally invasive and well tolerated without sedation. Particularly in patients with an indication for CE, it enables dual-organ evaluation in a single exam. This advantage possibly allows sparing of specialized resources and time, with the potential to reduce logistical burden.

Regarding safety, findings were consistent with the overall safety of CE, with one significant adverse event reported. Capsule retention is an uncommon adverse event of CE that is predictable in selected high-risk populations and can be prevented with a preceding patency capsule test [14,17]. Actually, RCE has an intermediate size (30.0 mm × 11.5 mm) between small bowel CE (26.0 mm × 11.4 mm) and CCE (32.3 mm × 11.6 mm).

**Figure 3:**
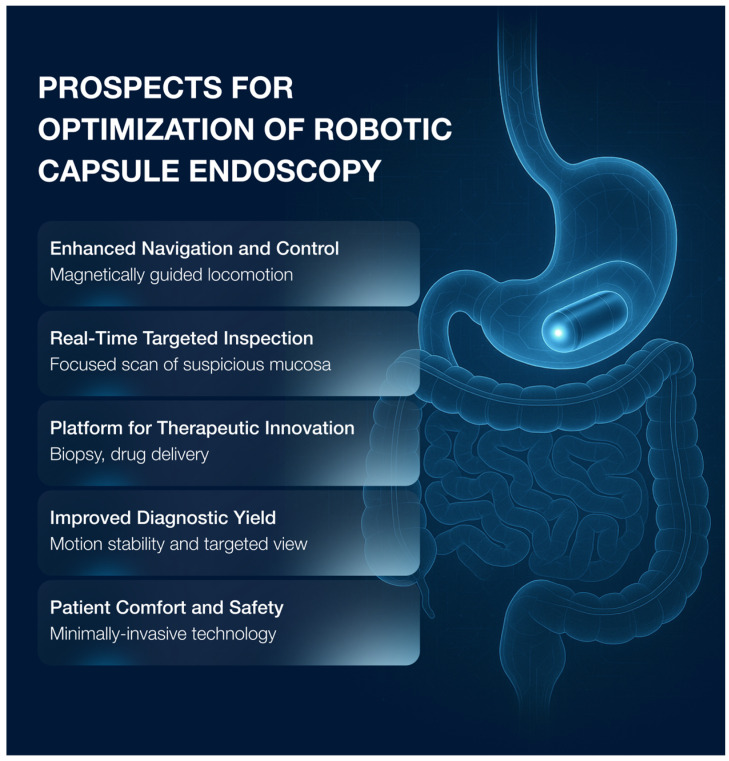
Prospects for optimization of robotic capsule endoscopy.

We must acknowledge that this interesting technique implies the installation of large hardware and the full-time presence of a trained health professional, which hinders its widespread deployment and first-line use for population-based screening. At the same time, this innovative guided navigation expands future possibilities of CE, from real-time targeted inspection to a platform for therapeutic intervention (Figure 3).

Currently, panendoscopy with CCE has the potential to be more accessible, including the possibility of at-home delivery. For long-term, artificial intelligence is expected to be instrumental in the extensive implementation of panendoscopy in clinical practice. Recent studies have demonstrated good accuracy of deep learning algorithms in the automatic detection of pleomorphic gastric lesions, a necessary step that is decisive for the development of a robust panendoscopic model [18,19,20,21,22,23,24].

RCE could be especially suitable for patients with a low pre-test probability of requiring endoscopic therapy or targeted biopsies through EGD, who are unfit or unwilling to undergo invasive procedures. Besides patient preference, it may also be particularly useful for outreach clinics where EGD with sedation is not regularly available, thereby preventing long travels. These findings support the integration of RCE into diagnostic pathways for patients requiring simultaneous gastric and small bowel assessment.

## Figures and Tables

**Table 1:** Patient demographic and clinical characteristics.

	Total Population (*n* = 85)
Age, years	
Median (IQR)	49 (40–64)
Range	25–79
Sex, *n* (%)	
Female	44 (52%)
Male	41 (48%)
BMI category, *n* (%)	
18.5–25 kg/m^2^	58 (68%)
25–30 kg/m^2^	23 (27%)
30–35 kg/m^2^	4 (5%)
Gastrointestinal surgery, *n* (%)	
Jejunal segmental resection	4 (5%)
Ileal segmental resection	2 (2%)
Right colectomy, ileocolonic anastomosis	2 (2%)
Total colectomy, ileorectal anastomosis	1 (1%)
Rectum anterior resection	1 (1%)
Main indication for SB CE, *n* (%)	
Suspected CD	30 (35%)
CD surveillance	19 (22%)
Iron deficiency anemia	26 (31%)
SB cancer surveillance	4 (5%)
Suspected SB cancer	2 (2%)
Hereditary polyposis syndrome	3 (4%)
Neurofibromatosis	1 (1%)
Main indication for gastroscopy, *n* (%)	
Suspected CD	21 (25%)
CD surveillance	17 (20%)
Dyspepsia	8 (%)
Polyp surveillance	8 (9%)
Angiectasia surveillance	6 (7%)
Chronic gastritis surveillance	7 (8%)
Iron deficiency anemia	7 (8%)
Hereditary polyposis syndrome	4 (5%)
Cancer screening	7 (8%)

IQR—interquartile range; CE—capsule endoscopy; CD—Crohn’s disease; SB—small bowel.

**Table 2:** RCE procedure details and main endoscopic findings.

	Total Population (*n* = 85)
Gastric transit time, minutes	
Median (IQR)	74 (35–106)
Small bowel transit time, minutes	
Median (IQR)	307 (253–388)
Gastric preparation, *n* (%)	
Good	32 (38%)
Fair	51 (60%)
Poor	2 (2%)
Small bowel preparation, *n* (%)	
Excellent	1 (1%)
Good	9 (11%)
Fair	71 (84%)
Poor	4 (5%)
Gastric findings, *n* (%)	
Gastritis	16 (19%)
Polyps	15 (19%)
Antrum	3 (4%)
Body	4 (5%)
Fundus	2 (2%)
Cardia	1 (1%)
Two or more Gastric regions	5 (6%)
Angiectasias	7 (8%)
Antrum/GAVE	4 (5%)
Body	1 (1%)
Fundus	2 (2%)
Diverticulum (fundus)	1 (1%)
Subepithelial lesion (antrum)	1 (1%)
Small bowel findings, *n* (%)	
P3 active bleeding	3 (4%)
P2 ulcers	15 (18%)
P2 angiectasias	14 (16%)
P2 tumors	3 (4%)
P2 varices	2 (2%)
P1 red spots	21 (25%)
P1 erosions	11 (13%)
P1 polyps	4 (5%)
P0 xanthelasmas	8 (9%)

IQR—interquartile range; GAVE—gastric antrum vascular ectasia.

## Data Availability

The data presented in this study are available on request from the corresponding author, and subject to data protection legislation.

## References

[1] Iddan G., Meron G., Glukhovsky A., Swain P. (2000). Wireless capsule endoscopy. Nature.

[2] Pennazio M., Rondonotti E., Despott E.J., Dray X., Keuchel M., Moreels T., Sanders D.S., Spada C., Carretero C., Valdivia P.C. (2023). Small-bowel capsule endoscopy and device-assisted enteroscopy for diagnosis and treatment of small-bowel disorders: European Society of Gastrointestinal Endoscopy (ESGE) Guideline—Update 2022. Endoscopy.

[3] Cardoso H., Rodrigues J.T., Marques M., Ribeiro A., Vilas-Boas F., Santos-Antunes J., Rodrigues-Pinto E., Silva M., Maia J.C., Macedo G. (2015). Malignant Small Bowel Tumors: Diagnosis, Management and Prognosis. Acta Med. Port..

[4] Santos-Antunes J., Cardoso H., Lopes S., Marques M., Nunes A.C.R., Macedo G. (2015). Capsule enteroscopy is useful for the therapeutic management of Crohn’s disease. World J. Gastroenterol..

[5] Eliakim R., Fireman Z., Gralnek I., Yassin K., Waterman M., Kopelman Y., Latcher J., Koslowsky B., Adler S.N. (2006). Evaluation of the PillCam Colon capsule in the detection of colonic pathology: Results of the first multicenter, prospective, comparative study. Endoscopy.

[6] Vuik F.E.R., Moen S., Nieuwenburg S.A.V., Schreuders E.H., Kuipers E.J., Spaander M.C.W. (2021). Applicability of colon capsule endoscopy as pan-endoscopy: From bowel preparation, transit, and rating times to completion rate and patient acceptance. Endosc. Int. Open.

[7] Rey J.F., Ogata H., Hosoe N., Ohtsuka K., Ogata N., Ikeda K., Aihara H., Pangtay I., Hibi T., Kudo S. (2012). Blinded nonrandomized comparative study of gastric examination with a magnetically guided capsule endoscope and standard videoendoscope. Gastrointest. Endosc..

[8] Shaukat A., Wang A., Acosta R.D., Bruining D.H., Chandrasekhara V., Chathadi K.V., Eloubeidi M.A., Fanelli R.D., Faulx A.L., ASGE Standards of Practice Committee (2015). The role of endoscopy in dyspepsia. Gastrointest. Endosc..

[9] Rey J.F. (2025). Magnetically guided gastric capsule endoscopy: A review and new developments. Clin. Endosc..

[10] Xiao Y.F., Wu Z.X., He S., Zhou Y.Y., Zhao Y.B., He J.L., Peng X., Yang Z.X., Lv Q.J., Yang H. (2021). Fully automated magnetically controlled capsule endoscopy for examination of the stomach and small bowel: A prospective, feasibility, two-centre study. Lancet Gastroenterol. Hepatol..

[11] Geropoulos G., Aquilina J., Kakos C., Anestiadou E., Giannis D. (2021). Magnetically Controlled Capsule Endoscopy Versus Conventional Gastroscopy: A Systematic Review and Meta-Analysis. J. Clin. Gastroenterol..

[12] Saurin J.C., Delvaux M., Gaudin J.L., Fassler I., Villarejo J., Vahedi K., Bitoun A., Canard J.M., Souquet J.C., Ponchon R. (2003). Diagnostic value of endoscopic capsule in patients with obscure digestive bleeding: Blinded comparison with video push-enteroscopy. Endoscopy.

[13] He C., Wang Q., Jiang X., Jiang B., Qian Y.Y., Pan J., Liao Z., Mascarenhas M., Cardoso H., Macedo G. (2023). Magnetic capsule endoscopy: Concept and application of artificial intelligence. Artificial Intelligence in Capsule Endoscopy: A Gamechanger for a Groundbreaking Technique.

[14] Sidhu R., Shiha M.G., Carretero C., Koulaouzidis A., Dray X., Mussetto A., Keuchel M., Spada C., Despott E.J., Zammit S.C. (2025). Performance measures for small-bowel endoscopy: A European Society for Gastrointestinal Endoscopy (ESGE) Quality Improvement Initiative—Update 2025. Endoscopy.

[15] Nass K.J., Zwager L.W., van der Vlugt M., Dekker E., Bossuyt P.M.M., Ravindran S., Thomas-Gibson S., Fockens P. (2022). Novel classification for adverse events in GI endoscopy: The AGREE classification. Gastrointest. Endosc..

[16] Brotz C., Nandi N., Conn M., Daskalakis C., DiMarino M., Infantolino A., Katz L.C., Schroeder T., Kastenberg D. (2009). A validation study of 3 grading systems to evaluate small-bowel cleansing for wireless capsule endoscopy: A quantitative index, a qualitative evaluation, and an overall adequacy assessment. Gastrointest. Endosc..

[17] Silva M., Cardoso H., Macedo G. (2017). Patency Capsule Safety in Crohn’s Disease. J. Crohns Colitis.

[18] Ribeiro T., Fernández-Urien I., Cardoso H., Mascarenhas M., Cardoso H., Macedo G. (2023). Colon capsule endoscopy and artificial intelligence: A perfect match for panendoscopy. Artificial Intelligence in Capsule Endoscopy: A Gamechanger for a Groundbreaking Technique.

[19] Mascarenhas M., Martins M., Afonso J., Ribeiro T., Cardoso P., Mendes F., Andrade P., Cardoso H., Ferreira J., Macedo G. (2023). The Future of Minimally Invasive Capsule Panendoscopy: Robotic Precision, Wireless Imaging and AI-Driven Insights. Cancers.

[20] Mascarenhas M., Mendes F., Ribeiro T., Afonso J., Cardoso P., Martins M., Cardoso H., Andrade P., Ferreira J., Mascarenhas Saraiva M. (2023). Deep Learning and Minimally Invasive Endoscopy: Automatic Classification of Pleomorphic Gastric Lesions in Capsule Endoscopy. Clin. Transl. Gastroenterol..

[21] Mota J., Almeida M.J., Mendes F., Martins M., Ribeiro T., Afonso J., Cardoso P., Cardoso H., Andrade P., Ferreira J. (2024). A Comprehensive Review of Artificial Intelligence and Colon Capsule Endoscopy: Opportunities and Challenges. Diagnostics.

[22] Mascarenhas M., Martins M., Afonso J., Ribeiro T., Cardoso P., Mendes F., Andrade P., Cardoso H., Mascarenhas-Saraiva M., Ferreira J. (2024). Deep learning and capsule endoscopy: Automatic multi-brand and multi-device panendoscopic detection of vascular lesions. Endos Int. Open.

[23] Saraiva M.J.M., Almeida M.J., Martins M., Afonso J., Ribeiro T., Cardoso P.M.M.S., Mendes F.M.C.S., Mota J., Andrade A.P., Cardoso H. (2025). Deep learning and capsule endoscopy: Automatic panendoscopic detection of protruding lesions. BMJ Open Gastroenterol..

[24] Martins M., Mascarenhas M.J., Almeida M.J., Afonso J., Ribeiro T., Cardoso P., Mendes F., Mota J., Andrade P., Cardoso H. (2025). A ubiquitous and interoperable deep learning model for automatic detection of pleomorphic gastroesophageal lesions. Sci. Rep..

